# Measuring the quality of mental health services from the patient perspective in China: psychometric evaluation of the Chinese version of the World Health Organization responsiveness performance questionnaire

**DOI:** 10.1080/16549716.2022.2035503

**Published:** 2022-03-15

**Authors:** Wei Zhou, Shuiyuan Xiao, Caixia Feng, Yu Yu, Dan Wang, Cheng Hu, Xiang Liu

**Affiliations:** a Research Center for Public Health and Social Security, School of Public Administration, Hunan University, Changsha, China; b Department of Social Medicine and Health Management, Xiangya School of Public Health, Central South University, Changsha, China; c Teaching Department, Liuzhou People’s Hospital, Liu Zhou, China; d Division of Prevention and Community Research & the Consultation Center, Department of Psychiatry, Yale School of Medicine, New Haven, Connecticut, USA; e Department of Public Health, Zhuzhou Central Hospital, Zhuzhou Central Hospital, Zhuzhou, China

**Keywords:** Mental health services, service quality, patient experiences, the responsiveness performance questionnaire, psychometric evaluation

## Abstract

**Background:**

Despite the large population of patients with mental disorders and the rapid development of mental health services in China, there are few evaluations of Chinese mental health services from the patient perspective. Relevant instruments with robust psychometric properties are lacking.

**Objective:**

This study aimed to translate, adapt and validate the WHO responsiveness performance questionnaire for measuring the quality of hospital mental health services among Chinese patients.

**Methods:**

The adaption of the translated questionnaire incorporated experts’ and patients’ opinions. For psychometric testing, 193 outpatients and 168 inpatients completed outpatient and inpatient modules, respectively.

**Results:**

The adapted questionnaire adhered to the WHO framework of responsiveness domains, and just four items had some wording changes. Item missing rates were below 6%. Both the outpatient and inpatient modules had acceptable internal reliability (Cronbach’s α = 0.837 and 0.730) and most domains had desirable average inter-item correlation coefficients. The confirmatory factor analysis indicated an acceptable model fit for the inpatient module, while some goodness-of-fit indices for the outpatient module were a little outside of the recommended ranges. Except for ‘talking privately’ from the domain of confidentiality (both outpatient and inpatient modules) and ‘waiting time’ from the domain of prompt attention (the inpatient module), factor loadings of all other items were above 0.5.

**Conclusions:**

The Chinese version of the responsiveness performance questionnaire has acceptable feasibility, reliability, and validity in general and it can be used to measure, assess and improve the quality of mental health services in China.

## Background

As patient-centred approaches have become widely recommended and used in healthcare delivery [1,2], patients’ perception has also become a vital source of evidence in healthcare evaluation and quality improvement [3,4]. Incorporating the patient perspective is of particular importance for evaluating mental health services because the chronic and relapsing nature of mental disorders needs positive patient experiences to promote adherence to treatment lasting for years [5,6].

The World Health Organization (WHO) has identified health, responsiveness and financing fairness as the three goals of health systems in the World Health Report 2000 and ‘responsiveness’ addresses patient experiences in contact with a healthcare system [7,8]. According to the WHO, ‘responsiveness’ measures a healthcare system’s performance of responding to users’ legitimate expectations regarding non-medical aspects of the service, apart from improvements in health or wealth [7,9]. The concept is different from satisfaction, one of the most commonly used indicators for measuring the quality of health services [10]. Patient satisfaction is a complex mixture of healthcare’s medical aspects (like treatment efficacy) and non-medical aspects (like interpersonal interactions and quality of amenities), and it can be influenced by medical aspects, like diagnoses and health outcomes [11,12]. Focusing on non-medical aspects, responsiveness attempts to measure what actually happens to patients and it describes healthcare quality without considering ‘whether health is directly improved by an encounter with the health system’ [13]. The WHO defines eight domains of responsiveness: dignity, confidentiality, communication, autonomy, choice, social support, quality of basic amenities, and prompt attention [13].

The responsiveness performance questionnaire, which is an operationalized instrument for measuring responsiveness, was developed from the literature review and expert opinions [13]. Field tests have demonstrated that the questionnaire has a good feasibility, reliability and validity [9,13]. To date, the questionnaire has not only been widely used in the WHO-led international surveys to assess health systems’ performance but has also been applied to evaluations of local general health services [14–16]. Researchers from Germany and Iran have also employed an adapted questionnaire to evaluate local mental health services [6,17].

The application of the responsiveness performance questionnaire as a quality indicator facilitates gathering patients’ feedbacks on health services [8]. After identifying poor performance of service provision, actions are expected to effect institutional change or system reform for improvement. For example, policy makers in many European countries have begun to introduce reforms to improve responsiveness of healthcare systems through important strategies, like addressing the issue of waiting lists and introducing or enlarging the choice of provider and purchaser [18]. In the last decade, there has also been increasing research attention on how to promote and utilize responsiveness as feedback loops between users and providers at the meso-level (organization/facility) or for specific services or programs [8]. Actions, like information systems and shared decision-making, have been proposed throughout service planning and provision [8,19–22].

In China, there are few evaluations of mental health services from the patient perspective [23]. Most of the few studies are patient satisfaction evaluations, which have reported a rather high level of satisfaction [23–25]. For example, a survey conducted in 32 tertiary psychiatric hospitals investigated inpatients’ satisfaction with communication, privacy protection, medical services and hospitalization costs, and a mean score of 23.3 ± 2.4 out of 25 was reported [23]. Another survey among patients discharged between 2015 and 2018 from a psychiatric hospital in Hebei Province reported patient satisfaction rates of over 97.5% on all assessed aspects, including psychiatrists’ attitudes and communication, medical ethics, medical techniques and devices, hospital environment and food, and hospitalization costs [24]. Another similar survey included 4063 discharged patients from a psychiatric hospital in Hubei Province reported an overall patient satisfaction rate of 95.7%, regarding medical treatment, clinical nursing, hospital environment and food, and hospitalization costs [25]. The instruments used in the existing evaluations are mainly self-designed. Lack of an established framework and psychometric testing is likely to cause potential reporting bias. In addition, different numbers of items and scoring methods also make comparison across institutions and regions difficult.

To address this gap, we aimed to translate, adapt and validate a Chinese version of the WHO responsiveness performance questionnaire for measuring the quality of mental health services among Chinese patients. Furthermore, unlike the German and Farsi versions which added new domains or divided and combined original domains [6,26], we adhered to the WHO eight-domain framework and made minimal adaption of each item, in order to facilitate cross-country comparisons between mental and general health services.

## Methods

### Study setting

This study setting was China’s mental healthcare system. In China, there are estimated 173 million adults with mental disorders, of whom 5.8 million are registered as having severe mental health problems [27,28]. In response to the increasing prevalence of mental disorders, large treatment gaps and high burdens of disease [29], the Chinese government has placed increasing priority on establishing and strengthening the mental healthcare system, which consists of hospital-based specialist services and community-based follow-up interventions for psychosis [30,31]. By the end of 2017, psychiatric hospitals and beds in China had increased to 1,170 and 445,000 respectively, and outpatient visits and inpatient discharges reached 37.5 million person-times and 1.98 million person-times, respectively [32]. Over 4.3 million patients with psychosis have been provided with regular follow-up services by primary health care staffs [28]. The focus areas for this study were outpatient and inpatient services provided by hospitals.

### Study design and data collection

This study adopted a two-phase design. The first phase was translation and adaption of the WHO responsiveness performance questionnaire. The second phase involved undertaking psychometric tests of the Chinese-version questionnaire for hospital mental health services through a cross-sectional study, which was part of an evaluation program on responsiveness in the mental healthcare system in China. This study was approved by the Institutional Review Board of the Xiangya School of Public Health, Central South University (XYGW-2018-01).

#### Phase I: Translation and adaption of the responsiveness performance questionnaire

The Chinese version of the responsiveness performance questionnaire for hospital mental health services was adapted based on the WHO original questionnaires used in the World Health Survey (WHS) and the Multi-country Survey Study (MCSS). The WHS version had 13 items for outpatient and home care and fifteen for inpatient care. The MCSS version had 22 items for ambulatory care and eleven for inpatient care [33]. All items from both the WHS and MCSS versions were translated from English to Chinese by one author (WZ) and then back translated by another author (YY), who were bilingual postdoctoral fellows of public mental health. The item translation was submitted to one bilingual professor of public health and also psychiatrist (SYX) for discrepancy or mistake identification and correction. After that, the research group (WZ, YY and SYX) discussed the item selection and wording adjustment based on China’s sociocultural context, the psychiatric setting and the need to remain colloquial. The selected and adjusted 13 items for the outpatient module and 15 items for inpatient module were then independently reviewed by four other researchers (two professors of social medicine and health management, one senior psychiatrist, and one senior nurse) and eight patients with mental disorders. The eight patients were recruited from outpatient (n = 3) and inpatient (n = 5) departments of the study sites, but they were not included in the main study. The research group reviewed feedbacks from the four researchers and eight patients and finalized the questionnaire.

The finalized Chinese-version questionnaire for hospital mental health services included seven domains with 13 items for the outpatient module and eight domains with 15 items for the inpatient module (Table 1). All items were rated on a 5-point Likert scale ranging from 1 = ‘very poor’/‘never’/‘extremely difficult’ to 5 = ‘very good’/‘always’/‘no difficult. Participants were asked to rate each item, based on their experiences of outpatient mental health services in the past 1 year or experiences of inpatient mental health services in the past 3 years.

**Table 1.** The Chinese version of the responsiveness performance questionnaire for hospital mental health services

Domains	Brief description of items	Selection sources
Dignity	Q1. When you received outpatient/inpatient mental health services, how would you rate your experience of being treated with respect? ^a^	WHS and MCSS
	Q2. When you received outpatient/inpatient mental health services, how would you rate the way your privacy was protected during physical examination and treatments? ^a^	WHS and MCSS
Confidentiality	Q3. When you received outpatient/inpatient mental health services, how often were your talks with mental health professionals done privately, without overhearing? ^b^	WHS and MCSS
	Q4. How would you rate the way your personal information was kept confidential by mental health professionals? ^a^	WHS and MCSS
Communication	Q5. How often did mental health professionals communicate things in a way you could understand ? ^b^	WHS and MCSS
	Q6. When you received outpatient/inpatient mental health services, how often did mental health professionals give you enough time to ask questions? ^b^	WHS and MCSS
Autonomy	Q7. How often did mental health professionals provide you with information of other types of treatments or tests? ^b^	WHS
	Q8. How often did mental health professionals ask your opinions when making decision related to treatment? ^b^	WHS and MCSS with wording change
Choice	Q9. How often could you choosing hospitals or clinicians you were happy with for appointment (outpatient)/for hospitalization (inpatient) ^b^	WHS and MCSS with wording change
Quality of basic amenities	Q10. How would you rate the cleanliness of the place (including toilets) of the outpatient mental health department/ of the inpatient ward? ^a^	WHS and MCSS
	Q11. How would you rate the overall comfortableness of the outpatient mental health department/ the inpatient ward? ^a^	MCSS with wording change
Prompt attention	Q12. How would you rate your travelling time to the hospital with an outpatient/inpatient mental health department? ^a^	WHS
	Q13. How would you rate your waiting time for making appointments and being attended at an outpatient mental health department/for being admitted to an inpatient mental health department? ^a^	WHS with wording change
Social support*	Q14. How difficult was it to have your family and friend visits during your hospitalization in an inpatient mental health department? ^c^	WHS
	Q15. How difficult was it to stay in contact with the outside during your hospitalization in an inpatient mental health department? ^c^	WHS

^a^Response options: very poor (1), poor (2), moderate (3), good (4), very good (5)

^b^Response options: never (1), seldom (2), sometimes (3), usually (4), always (5)

^c^Response options: extremely difficult (1), difficult (2), moderate (3), little difficult (4), no difficulty (5)

*The domain of social support and its items were not included in the outpatient module.

#### Phase II: Psychometric evaluation

Patients were eligible for the study if they: (i) were older than 18 years of age; (ii) had been diagnosed with any type of mental disorders under the International Statistical Classification of Diseases and Related Health Problems-10th revision (ICD-10); (iii) were mentally capable of understanding interviews according to medical staff’s judgment or their clinical records, and (iv) had received outpatient or inpatient mental services in the past 1 year. Patients treated for substance abuse were excluded. According to ‘the rule of thumb’, at least 10 participants are required for each scale item [34]. We calculated a minimal sample size of 130 outpatients and 150 inpatients.

(1) The outpatient module survey: An online survey was conducted using a convenience sample of patients recruited from the outpatient mental health department in a large general tertiary hospital in Changsha, the capital city of Hunan Province. Nurses at the mental health department helped identify returning adult patients and judge their mental capability through short conversations. A study information sheet with the Quick Response Code of the online survey was distributed to each eligible patient. Patients, who were willing to participate, could use their own mobile phones to scan the Quick Response Code and complete the survey. The survey collected patients’ social-demographic characteristics, diagnosis, service-utilization related information and responses to the responsiveness performance questionnaire. The survey was conducted between 3 September and 5 November 2018. Responses were anonymous, and patients could withdraw anytime throughout the survey.

(2) The inpatient module survey: A face-to-face survey and a telephone follow-up interview were conducted among patients discharged between 1 June and 31 July 2018 from the inpatient mental health department in another large general tertiary hospital in Changsha. After obtaining written consent, eligible patients were interviewed face to face either on the day or one day before patient discharge. The following data were collected: (i) social-demographic characteristics; (ii) discharge diagnosis and service-utilization related information; (iii) telephone number and call time preference for the follow-up survey. To minimize the influence of hospitalization status on patients’ ratings [35], the responsiveness performance questionnaire was administered in the follow-up telephone survey, which was completed within 1 week after patient discharge. To increase the response rate, advance text messages notifying the upcoming telephone interview were sent to each participant on the day of calling. Both face-to-face and telephone surveys were conducted by postgraduate students with a background in public health.

### Data analysis

In accordance with the WHO, we used a standard set of feasibility, reliability and validity tests to evaluate psychometric properties of the Chinese version of the responsiveness performance questionnaire [9]. Feasibility was tested by item missing rates: above 5% was considered as problematic. Responses of ‘unknown’, ‘refuse’ or ‘not applicable’ were all treated as missing data. Cronbach’s alpha coefficients and average inter-item correlation coefficients were used to assess internal reliability. Acceptable Cronbach’s alpha coefficients were set to be 0.7 or greater [36]. As Cronbach’s alpha coefficients are sensitive to the number of items and increase with increased item numbers, we only calculated the average inter-item correlation coefficients for each domain. These were judged as optimal if between 0.3 and 0.8 [37,38].

Confirmatory factor analysis was conducted to assess the construct validity of the Chinese-version questionnaire under the established factor structure by the WHO. The loading of the only item under the domain of choice was fixed to 1.0. The model fit was assessed using the root mean square error of approximation (RMSEA), comparative fit index (CFI), Tucker-Lewis Index (TLI) and standardized root mean square residual (SRMR). An acceptable model fit was defined: RMSEA and SRMR < 0.08, and CFI and TLI > 0.90 [39,40]. We did not rely on χ2 test for its sensitivity to the sample size [39]. Standardized item loadings on factors of 0.3 were considered to be minimally significant, 0.4 were important, 0.5 were significant [26].

The item ratings were treated as interval data, as suggested by the WHO [9]. All analyses were performed in SPSS 23.0 and Mplus 7.4; statistical significance level was set as 0.05 and all tests were two-tailed.

## Results

### Questionnaire adaption

The Chinese version of the responsiveness performance questionnaire for hospital mental health services adhered to the WHO's original framework of domains, and only four items (Q8, Q9, Q11, and Q13) had wording changes (Table 1). For items Q8, Q9, Q11, the amended wording provided more specific definitions or information on the activities involved. For example, in the Q8, we used ‘asking patients’ opinions’ to replace the general description of ‘being involved in making decisions’ which was in the original questionnaire. The change was intended to improve understandability, which was based mainly on the feedback from patient participants in the pilot study. For a similar reason, the adapted Q9 specified the original ‘healthcare providers’ as ‘hospitals or clinicians’. We also provided a more specific scenario of choosing hospitals or clinicians by adding ‘for (outpatient) appointment/for (inpatient) hospitalization’. The adapted Q11 replaced the original term of ‘quality’ with ‘comfortableness’ for better clarification. For the item of Q13, the wording change had a special emphasis on the waiting time for ‘making appointments (outpatient)’ and ‘being admitted (inpatient)’, due to the difficulties of getting health services in China [41].

### Psychometric tests

#### (1) Participant characteristics

A total of 193 patients completed the outpatient module survey. For the inpatient module survey, 234 eligible patients agreed to participate and completed the face-to-face interviews. In the telephone follow-up interview, 29 were lost to follow-up due to having given wrong or invalid telephone numbers, 20 did not answer phone calls on three occasions, 12 refused to participate after answering the phone, five withdrew before finishing the survey, and 168 finished the responsiveness performance questionnaire. Among patients who answered phone, the response rate was 90.8%.

Table 2 lists participants’ social-demographic characteristics, diagnosis and service utilization information. The mean age of the outpatient participants was 27, and almost half were female. The majority of outpatient participants were diagnosed with mood (34.2%) or anxiety disorders (36.3%). Seventy-seven (39.9%) had received outpatient mental health services in multiple hospitals in the previous year. The mean age of the inpatient participants was 35 and just over half were female. Thirty-five patients (20.8%) were diagnosed with schizophrenia and other psychotic disorders, 81 (48.2%) with mood disorders, 31 (18.5%) with anxiety disorders and 21 (12.5%) with somatoform, eating or sleeping disorders. Around one third of the patients reported having received inpatient mental health services in different hospitals in the past 3 years.

**Table 2.** Characteristics of the outpatient and inpatient participants

Variables	Outpatients (n = 193)	Inpatients (n = 168)
Age, Mean(sd)	27.7 (8.4)	35.3 (14.0)
Male, n(%)	82 (42.5)	83 (49.4)
Marital status*, n(%)		
Married	65 (33.7)	90 (53.6)
Unmarried	128 (66.3)	78 (46.4)
Education, n(%)		
≤Primary school	6 (3.1)	12 (7.1)
Junior high school	22 (11.4)	39 (23.2)
Senior high school	40 (20.7)	49 (29.2)
≥University	125 (64.8)	68 (40.5)
Employment, n(%)		
Employed	96 (49.7)	69 (41.1)
Unemployed	97 (50.3)	99 (58.9)
Diagnosis, n(%)		
Schizophrenia and other psychotic disorders	19 (9.8)	35 (20.8)
Mood disorders	66 (34.2)	81 (48.2)
Anxiety disorders	70 (36.3)	31 (18.5)
Others**	33 (17.1)	21 (12.5)
Unknown	5 (2.6)	0 (0)
Treatment in different mental health facilities in the past one year (outpatient)/past three years (inpatient)	77(39.9)	50 (29.9)

*Married includes married and cohabited, unmarried includes single, divorced, and widowed.

**Others include somatoform disorders, eating disorders, and sleeping disorders.

#### (2) Feasibility

For the outpatient module, there were no missing data for any item. Automatic quality-control measures in the online survey meant that participants had to answer all questions before proceeding or submitting. For the inpatient module, only ‘waiting time’ from the domain of prompt attention was slightly above the cut-off missing rate of 5% (Table 3). This relatively high item missing rate was possibly because the domain of prompt attention was placed last in the telephone survey. The interviewers reported that some participants had become less patient in answering questions at this time.

**Table 3.** Psychometric tests of the outpatient and inpatient modules

		Item missing rate (%)	Average Inter-item correlation		Factor loadings	
Domain	Item	Inpatient	Outpatient	Inpatient	Outpatient	Inpatient
Dignity	Q1. Treating with respect	0	0.752	0.564	0.823	0.711
	Q2. Physical privacy protection	1.19			0.914	0.795
Confidentiality	Q3. Talking privately	1.79	0.256	0.281	0.297	0.357
	Q4. Keeping information confidential	1.19			0.863	0.786
Communication	Q5. Communication understandable	0	0.474	0.361	0.541	0.589
	Q6. Time for questioning	0.6			0.876	0.617
Autonomy	Q7. Information on alternatives	1.79	0.590	0.462	0.687	0.669
	Q8. Asking patients’ opinions	2.38			0.859	0.692
Choice	Q9. Choosing a healthcare provider	4.17	n/a	n/a	1.000	1.000
Quality of basic amenities	Q10. Cleanliness	2.41	0.625	0.536	0.812	0.733
	Q11. Overall comfortableness	2.29			0.770	0.732
Prompt attention	Q12. Travelling time	2.98	0.505	0.215	0.530	0.745
	Q13. Waiting time	5.95			0.954	0.290
Social Support	Q14. Family/friend visits	2.98	n/a	0.522	n/a	0.715
	Q15. Contacting outside	3.57			n/a	0.727

#### (3) Internal reliability

For the outpatient module, the overall Cronbach’s alpha coefficient was 0.837. The domain of confidentiality had the worst performance in the average inter-item correlation coefficient (r = 0.256). All other domains had average inter-item correlation coefficients ranging from 0.474 to 0.752 (Table 3).

For the inpatient module, the overall Cronbach’s alpha coefficient was 0.730. The domains of confidentiality (r = 0.281) and prompt attention (r = 0.215) were the worst performing domains in average inter-item correlation coefficients. Average inter-item correlation coefficients of the other domains were all above 0.3 (Table 3).

#### (4) Construct validity

Some goodness-of-fit indices were a little outside of the acceptable ranges for the outpatient module (RMSEA = 0.081, CFI = 0.938, TLI = 0.893, SRMR = 0.053). According to Table 3, ‘talking privately’ in the domain of confidentiality had the lowest factor loading of 0.297. Factor loadings of the other 12 items ranged from 0.530 to 0.964.

Fit indices of the model were acceptable for the inpatient module (RMSEA = 0.035, CFI = 0.973 TLI = 0.954, SRMR = 0.042). Factor loadings of ‘talking privately’ (Domain: Confidentiality) and ‘waiting time’ (Domain: Prompt attention) were as low as 0.357 and 0.290, respectively. Factor loadings of all other 13 items were above 0.5 (Table 3).

## Discussion

This study was the very first attempt to introduce an instrument to measure the quality of China’s hospital mental health services from the perspective of patients. The results demonstrated that the widely used responsiveness performance questionnaire was applicable to outpatient and inpatient services in the context of China’s mental healthcare system.

Although the Chinese version of the responsiveness performance questionnaire had acceptable psychometric properties in general, the domains of confidentiality and prompt attention performed less well. For the domain of confidentiality, the average inter-item correlation coefficients were below 0.3 in both outpatient and inpatient modules. In addition, the item of ‘talking privately’ did not perform well enough in the construct validity, with factor loadings of 0.297 in the outpatient module and 0.357 in the inpatient module. The low correlation between ‘talking privately’ and ‘keeping patient information confidential’ was also reported in psychometric tests of the responsiveness performance questionnaire for general hospital services and community mental health services in China [42,43]. A potential explanation given by previous studies is that clinicians could hardly talk privately with each patient due to the large patient volume and the over-crowdedness in Chinese medical institutions [42–44], which is also consistent with our site observation.

For the domain of prompt attention in inpatient module, the average inter-item correlation coefficient was 0.215, and the item of ‘waiting time’ had a low factor loading of 0.290 in the construct validity. The performance in this domain was also less satisfactory in the field tests of the MCSS version among general health patients across countries and the Farsi version among mental health patients from Iran. The wording and mixing of numerical and categorical response data were listed as primary explanations [13,26]. The Iranian researcher also proposed that the waiting time and distance were not intrinsically correlated [26]. Yet this is more applicable for our study in China. Patients with mental disorders may receive hospitalized treatment several times over the course of 3 years. The travelling time to hospitals can be relatively consistent or even fixed for individual patients. However, the waiting time for the patient’s admission can have more uncertainty, which is influenced by multiple factors, such as the severity of the patient’s condition and bed turnover rates within different hospitals and periods [45], especially within the context of overcrowded hospitals in China [44].

### Value and use of the research

This research has been a significant addition to the methodological literature on China’s mental health services, which provides a robust instrument for future evaluations. In addition, as responsiveness provides a universal quality indicator applicable to all concerned services [9,46], the Chinese-version questionnaire could not only be used to facilitate comparison of quality assessment between China and the global mental health community but also to detect gaps between general and mental health services in China. Ultimately, this can make a positive contribution to quality improvement in China’s mental healthcare system.

In China, health reforms are also moving towards person-centered care [47]. Incorporating the Chinese-version questionnaire in quality monitoring or routine surveys will accelerate the patient-oriented transformation of mental health services in China. Furthermore, low health service responsiveness is related to patients avoiding medical care despite being in need of it [48]. Previous studies also identified that having poor patient experiences was one important barrier to seeking mental health services in China [49], where only 24.1% of patients with mental disorders meet the minimum standards for adequacy of treatment [50]. Therefore, gathering patients’ feedbacks and providing mental health services with better responsiveness could promote help-seeking behaviours and thus improve population mental health in China.

### Limitations

In the study, we did not evaluate the questionnaire’s test–retest reliability for the following reasons. (i) For the outpatient module, no personal identified information was collected through the survey. This made implementing a retest survey infeasible. Even if the retest survey could be implemented, there was also concern regarding patients’ mental health, which is difficult to assess through an online retest survey. In addition, participants’ responses in the retest survey could be potentially influenced by their new experiences of outpatient mental health services between the initial and the retest surveys. (ii) For the inpatient module, the survey was conducted within 1 week following discharge when patient’s mental condition was expected to be relatively stable due to sustained influence of medication during the hospitalization [51]. As non-adherence is likely among patients with mental disorders [52], uncertain mental conditions would also be a particular concern for a retest survey 2 to 4 weeks post discharge, based on the WHO time interval between the initial and re-administered responsiveness performance interviews [9].

As the present study was part of an evaluation program on responsiveness of China’s mental healthcare system, the time frames of the Chinese-version questionnaire were set to be ‘the past one year’ in the outpatient module and ‘the past three years’ in the inpatient module, in an attempt to cover participants’ multiple experiences. However, we acknowledge that these time frames may result in recall bias. This study only provides a starting point for testing the Chinese version of the responsiveness performance questionnaire for hospital mental health services. Future studies are needed to explore the following issues: (i) how the instrument could be further refined, especially regarding the less well-performed domains of confidentiality and prompt attention, and (ii) whether psychometric properties of the questionnaire might vary across patient groups with different types of mental disorders and severity of conditions.

## Conclusions

The study translated, adapted and validated the WHO responsiveness performance questionnaire for measuring the quality of outpatient and inpatient mental health services among patients in China. The Chinese-version questionnaire includes 7 domains with 13 items for the outpatient module and 8 domains with 15 items for the inpatient module. The results of these psychometric tests demonstrated the instrument’s acceptability, feasibility, reliability and validity in general. The future application of the questionnaire in China’s mental health services assessment will make a positive contribution to the improvement of service quality and patient experiences.

## Paper context

Few evaluations on China’s mental health services are from the patient perspective. Relevant instruments with robust psychometric properties are lacking. This study translated, adapted and psychometrically tested a WHO standardized instrument for measuring patient-reported quality of hospital mental health services in China. The results demonstrate that the Chinese version of the responsiveness performance questionnaire has acceptability, feasibility, reliability and validity. This questionnaire can be used to measure the quality of mental health services in China and facilitate comparison across countries.
